# A Novel Pulsatile Bioreactor for Mechanical Stimulation of Tissue Engineered Cardiac Constructs

**DOI:** 10.3390/jfb2030107

**Published:** 2011-07-20

**Authors:** Trixi Hollweck, Bassil Akra, Simon Häussler, Peter Überfuhr, Christoph Schmitz, Stefan Pfeifer, Markus Eblenkamp, Erich Wintermantel, Günther Eissner

**Affiliations:** 1 Department of Cardiac Surgery, University of Munich, Marchioninistrasse 15, 81377 Munich, Germany; E-Mails: Trixi.Hollweck@med.uni-muenchen.de (T.H.); Bassil.Akra@med.uni-muenchen.de (B.A.); Simon_Haeussler@web.de (S.H.); Peter.Ueberfuhr@med.uni-muenchen.de (P.U.); Christoph.Schmitz@med.uni-muenchen.de (C.S.); 2 Chair of Medical Engineering, Technische Universität München, Boltzmannstrasse 15, 85748 Garching, Germany; E-Mails: Pfeifer@medtech.mw.tum.de (S.P.); Eblenkamp@medtech.mw.tum.de (M.E.); Wintermantel@medtech.mw.tum.de (E.W.)

**Keywords:** tissue engineering, bioreactor, mechanical stimulation, mesenchymal stem cells, cardiac differentiation, umbilical cord

## Abstract

After myocardial infarction, the implantation of stem cell seeded scaffolds on the ischemic zone represents a promising strategy for restoration of heart function. However, mechanical integrity and functionality of tissue engineered constructs need to be determined prior to implantation. Therefore, in this study a novel pulsatile bioreactor mimicking the myocardial contraction was developed to analyze the behavior of mesenchymal stem cells derived from umbilical cord tissue (UCMSC) colonized on titanium-coated polytetrafluorethylene scaffolds to friction stress. The design of the bioreactor enables a simple handling and defined mechanical forces on three seeded scaffolds at physiological conditions. The compact system made of acrylic glass, Teflon^®^, silicone, and stainless steel allows the comparison of different media, cells and scaffolds. The bioreactor can be gas sterilized and actuated in a standard incubator. Macroscopic observations and pressure-measurements showed a uniformly sinusoidal pulsation, indicating that the bioreactor performed well. Preliminary experiments to determine the adherence rate and morphology of UCMSC after mechanical loadings showed an almost confluent cellular coating without damage on the cell surface. In summary, the bioreactor is an adequate tool for the mechanical stress of seeded scaffolds and offers dynamic stimuli for pre-conditioning of cardiac tissue engineered constructs *in vitro*.

## 1. Introduction

For the use of tissue engineered constructs *in vivo*, it is essential to examine their functionality and mechanical integrity prior to implantation [1]. In addition, forces acting directly or indirectly on cells, via scaffolds for example, can affect cellular differentiation [2]. *In vivo*, cells are stimulated continuously by mechanical, electrical and chemical signals influencing their phenotype, morphology and proliferation. If these signals are inappropriate or absent, cells lose their ability to form organized tissue [3]. Thus, bioreactors simulating physiological conditions, such as mechanical shear stress, play a crucial role in the development of tissue engineered constructs [1]. The development of an effective bioreactor requires the consideration of various parameters. Ideally, bioreactors allow the regulation of physical parameters such as temperature, pH, pO_2_, pCO_2_, allow nutrient supply and removal of toxic metabolites as well as mechanical stimuli. Moreover, the material must be compatible with the manufacturing process, sterilization technique and the cultured cell type [4]. Bioreactors can be distinguished by their application: bioreactors for cell seeding, cultivation of colonized scaffolds and for conditioning of functional tissue engineered prostheses [5,6,7]. In heart valve fabrication, the development of bioreactors for tissue formation under dynamic culture conditions was demonstrated several times [1,8,9]. It is also already known, that bioreactors support tissue formation of heart muscle *in vitro* [10,11]. An effective approach to improve the contractile properties of artificial heart muscle constructs is electrical field stimulation or mechanical stimulation by unidirectional or auxotonic stretching [12]. Accompanied by an improvement of contractile function, some studies demonstrated extracellular matrix formation, increased cell proliferation and uniform cell distribution of strained constructs [13,14]. In this context, Zimmermann *et al.* reported from highly differentiated cardiac tissue constructs after cyclic mechanostimulation in a stretch device [15]. For the fabrication of an autologous patch tissue for cardiovascular surgery, Sodian *et al.* developed a closed-looped perfused bioreactor by combining pulsatile perfusion and periodically stretching of tissue-engineered patch constructs [16]. Birla *et al.* described a bioreactor system that applies electromechanical stretch to bioengineered heart muscle constructs with good results and no evidence of physical damage [17]. In order to repopulate ischemic myocardium with cells that could restore contractility, we previously demonstrated that titanium-coated clinically approved cardiovascular patches enhance retention of human umbilical cord tissue derived mesenchymal stem cells (UCMSC) and thus offer a potential cell delivery system for the repair of damaged myocardium [18]. In addition to the static seeding procedure described in [18], the aim of the present study was to analyze the stability of the cellular coating upon mechanical stress in a newly developed bioreactor mimicking myocardial contraction.

## 2. Experimental Section

### 2.1. Bioreactor Construction

The bioreactor was designed using the CATIA V5R19 software (IndustrieHansa Consulting & Engineering GmbH, München, Germany). Bioreactor components were manufactured in-house. The core unit of the bioreactor, consisting of media compartments (outer diameter D = 20 mm, inner diameter d = 10 mm, outer height H = 115 mm, inner height h = 100 mm), sample compartment (D = 135 mm, H = 20 mm), pressure compartment (D = 135 mm, d = 110 mm, H = 40 mm, h = 5 mm) and clip-systems (D = 23 mm, H = 6 mm; upper part: D = 23 mm, d = 9 mm, H = 5 mm, lower part: D = 20 mm, d = 11 mm, H = 3 mm) were produced from acrylic glass and polyvinylchloride (Sahlberg GmbH & Co. KG, München, Germany), respectively. Gaskets and pulse-membranes (D = 20 mm; thickness = 0.5 mm) were made of silicone (Sahlberg GmbH & Co. KG, München, Germany). Lining disks were produced from Teflon® (Sahlberg GmbH & Co. KG, München, Germany). Actuation parts (connection rod (55 × 7 × 5 mm), eccentric wheel (D = 50 mm, H = 5 mm), piston (D_p_ = 12 mm), piston rod (d = 8 mm, h = 50 mm), cylinder (D = 25 mm, d= 13 mm, H_c_ = 30 mm) were made of stainless steel (Inoxium Edelstahlhandel, Rosenheim, Germany); gear motor, speed controller and power supply were purchased from Modelcraft Inc. (Blaine, USA), H-Tronic GmbH (Hirschau, Germany) and Conrad Electronic SE (Hirschau, Germany), respectively.

### 2.2. Bioreactor Sterilization

**Figure 1:**
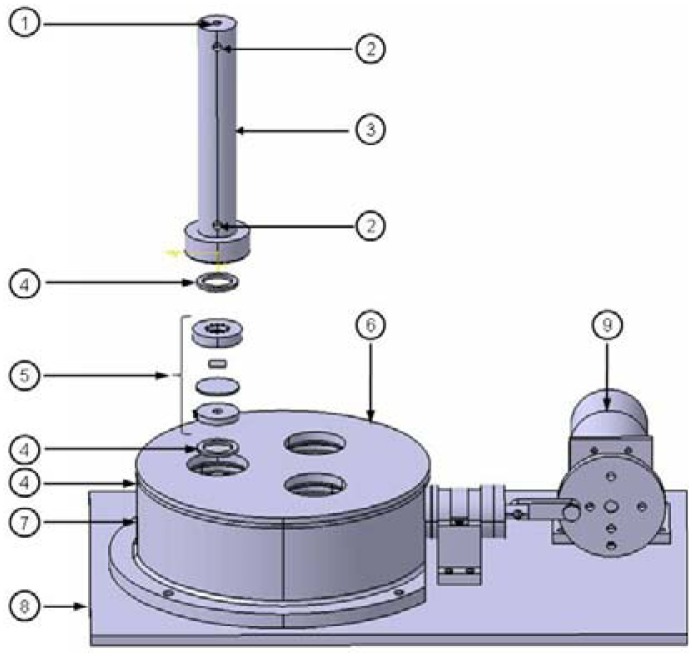
Schematic illustration of the novel pulsatile bioreactor. (1) Luer-lock-connector for CO_2_-gassing, (2) luer-lock-connectors for nutrient supply and removal, (3) media compartment, (4) gasket, (5) clip-system, (6) sample compartment, (7) pressure compartment, (8) base plate, (9) actuation unit. By generating a pulsatile pressure in the pressure compartment, pulse membranes in the clip-systems bend and seeded scaffolds, also fixed in the clip-system, are subjected to friction stress. Nutrient supply of seeded scaffolds was provided by medium in the media compartment. Physiological conditions are provided by integrating the whole bioreactor in a standard incubator. Sterile filters on the top of the three media compartments secure CO_2_-exchange. Scale bar = 20 mm.

The sterilization of the bioreactor was restricted to components in contact with cells and/or cell culture medium; in detail, the media compartments, sample compartment, clip-systems, pulse-membranes, gaskets and lining disks (see Figure 1). The sterilization was performed by formaldehyde deposition at 60–70 °C for 7 h and was evaluated during typical process conditions for 96 h in a standard incubator at 37 °C/5% CO_2_. Samples were aseptically taken at 24 h and 96 h and were screened for contaminations by conventional microbiological evaluation methods (Max-von-Pettenkofer-Institut für Hygiene und medizinische Mikrobiologie, University of Munich, Germany).

### 2.3. Bioreactor Functionality

Bioreactor functionality was checked by connecting a pressure transducer (Gould-Statham Inc., Oxnard, USA) and a pressure monitoring system (Siemens AG, München, Germany) to the pressure compartment of the bioreactor, in order to measure the produced pulsating pressure. A further control parameter was the visualization of pulsating menisci in the media compartments.

### 2.4. Cell Seeding on Non-Degradable Synthetic Scaffolds

#### 2.4.1. Cell Type

Umbilical cord mesenchymal stem cells (UCMSC) derived from human umbilical cord tissue were used for scaffold seeding. Isolation, cell culture and phenotypic characterization of UCMSC were performed as previously described. Briefly, UCMSC were isolated according to Seshareddy *et al.* [19] and were cultured in complete xenofree medium. For phenotypic characterization, UCMSC were stained for mesenchymal and hematopoetic markers and were analyzed by flow cytometry [20]. As a functional control, UCMSC were differentiated into the osteogenic and adipogenic lineage and were histochemically analyzed [21,22].

#### 2.4.2. Scaffold Type

As previously described, UCMSC were seeded on clinically approved Cardiovascular Patch (CVP) composed of expanded Polytetrafluorethylene (ePTFE) [18]. Briefly, CVP with a thickness of 0.4 mm features a homogenous macroporous surface with a pore size of 22 μm and were kindly provided by W. L. Gore Associates GmbH (Putzbrunn, Germany). In order to increase surface energy, promoting electrostatic interactions with molecules and proteins, the scaffold was coated with titanium (pfm medical titanium gmbh, Nürnberg, Germany). For coating, titanium containing molecules are split into their atomic elements by plasma-assisted chemical vapor deposition (PACVD). The titanium atoms adhere to the entire surface of scaffolds, form a covalent chemical compound and constitute in contact with air, a titanium dioxide film resulting in an improved wettability. For fixing in the bioreactor, titanium-coated scaffolds were punched in the dimensions of 7.5 mm × 7.5 mm, tagged with a surgical suture and sterilized by steam at 121 °C for 20 min.

#### 2.4.3. Seeding Procedure

For reproducible seeding, commercial available inserts made of stainless steel and silicone (Aix scientifics® CRO, Aachen, Germany) were placed on the scaffold in 24-well-plates. These cylindric, so-called “Fences” were used to pre-define the dimension of the seeding area. For seeding, 3 × 10^2^ UCMSC/mm^2^ were introduced into the Fences according to the manufacturer's protocol and were incubated at 37 °C/5% CO_2_ for 72 h.

### 2.5. Adherence Rate of UCMSC

Adherence rates of UCMSC seeded on titanium-coated CVP were determined with or without mechanical stress. Mechanical stress tests were performed at 65 bpm for 24 h in a standard incubator at 37 °C/5% CO_2_. Control scaffolds without exposure to mechanical stress were incubated under static conditions in 24-well-plates for 24 h at 37 °C/5% CO_2_. Seeded scaffolds were fixed in methanol-acetone (1:2) for 2 minutes at RT and rinsed with PBS (PAA Laboratories GmbH, Pasching, Austria). Cell nuclei were stained with 1 μg/mL diamidinophenylindole (DAPI; Roche Diagnostics GmbH, Mannheim, Germany) for 20 minutes at RT in the dark. Samples were analyzed at 358 nm by fluorescence microscopy (Zeiss MicroImaging GmbH, Jena, Germany). The number of attached UCMSC was quantified by DAPI-stained cell nuclei, related to their initial seeding density and standardized to UCMSC without mechanical stress. Normalization was performed to exclude cord-donor specific deviations in cell adherence on scaffolds.

### 2.6. Scanning Electron Microscopy (SEM)

In order to assess the morphology of UCMSC under statical or mechanical conditions in the newly developed bioreactor at 65 bpm for 24 h, seeded scaffolds were fixed in 3% glutardialdehyde (Merck KGaA, Darmstadt, Germany) over night at 4 °C. Subsequently, scaffolds were washed with PBS and dehydrated by incubation in an afferent ethanol series (30%, 50%, 70%, and 90%) and subsequently in 3 × 100% acetone (Merck KGaA, Darmstadt, Germany). After sample drying at the critical point, scaffolds were sputtered with gold film for 180 s at 10^−5^ mbar and finally, analyzed microscopically by using a scanning electron microscope (EVO® LS10, Zeiss MikroImaging GmbH, Jena, Germany) and a SE-detector at 15 kV.

## 3. Results and Discussion

### 3.1. Bioreactor Design

As shown in Figure 1, the novel pulsatile bioreactor consists of a core unit with three media compartments (3), three clip-systems each with a pulse-membrane (5), a sample compartment (6), a pressure compartment (7) and an actuation unit (9).

#### a) Core Unit

The sterile, cylindric media compartments are separated from the unsterile sample compartment by pulse-membranes). Media compartments can be filled with media (maximal volume = 7.85 mL) via luer-lock-connectors (2). Pulse-membranes and seeded scaffolds are fixed in clip-systems. The clip-systems are fixed between the sample compartment and the media compartments. The sample compartment is screwed onto the cylindric pressure compartment, which is connected to the actuation unit.

#### b) Actuation Unit

**Figure 2:**
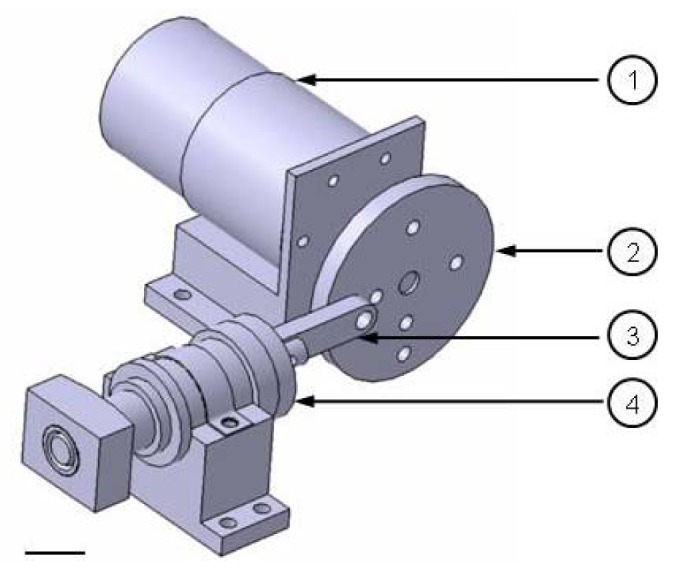
Actuation unit of the bioreactor. (**1**) Gear motor; (**2**) eccentric wheel; (**3**) connection rod; (**4**) cylinder with piston. The adjustable actuation unit generates a variable pulsatile pressure at frequencies of 1–65 bpm. Scale bar = 20 mm.

The actuation (see Figure 2) consists of an electric gear motor with adjustable speed, which moves a connection rod (3) by an eccentric wheel (2). The connection rod is bonded to a piston by a piston rod, resulting in a horizontal movement of the piston in a cylinder (4) and a displaced volume (V_p_ = (D_p_^2^ π/4) H_c_) of 3.4 cm^3^. The cylinder is connected to the pressure compartment to generate a variable pulsatile pressure at frequencies of 1–65 bpm.

### 3.2. Bioreactor Function

**Figure 3:**
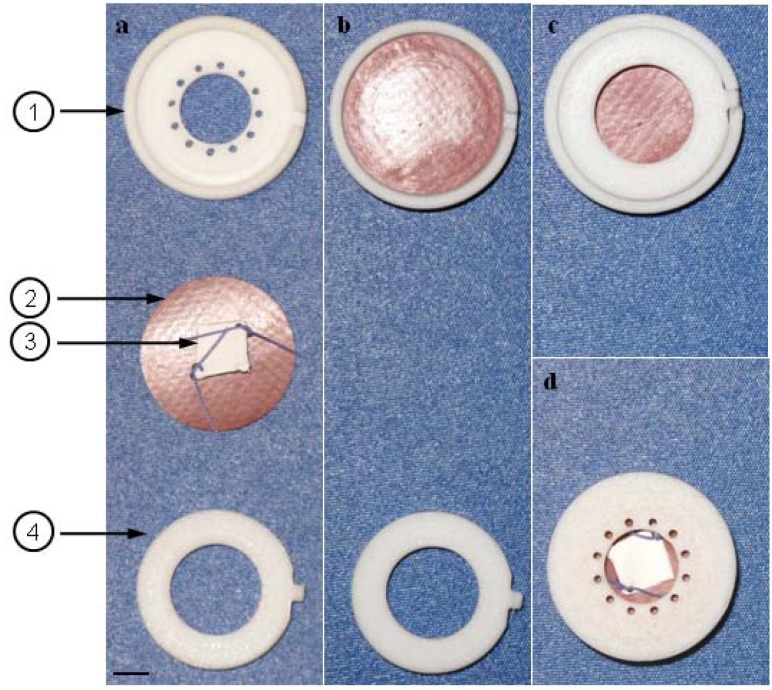
Clip-system for fixation of pulse-membrane and scaffold. (**1**) Upper part, (**2**) pulse-membrane, (**3**) seeded scaffold, (**4**) lower part. (**a**) The populated side of the scaffold is centered to the pulse-membrane. (**b**) The pulse-membrane is inserted into the upper part, arranging the scaffold between upper part and pulse membrane. (**c**) The system is locked by the lower part and (**d**) transferred backwards to the sample compartment of the bioreactor. Pulsating membranes result in friction stress of seeded scaffold. Scale bar = 0.4 cm.

By moving the piston, air is alternately compressed and relaxed in the pressure compartment. At piston end position, the pressure in the pressure compartment is maximal and the pulse-membranes in the clip-systems are bending to balance the pressure gradient. At piston start position, a pressure gradient does not exist and the pulse-membranes relax. As shown in Figure 3, pulse-membrane and seeded scaffold (3) are fixed in the sample compartment by a clip-system. Alternately bending and relaxing membranes simulate myocardial contraction, resulting in friction stress of seeded scaffolds. The clip-system consists of an upper part (1), a pulse-membrane (2) and a lower part (4). The populated side of the scaffold is centered to the pulse-membrane (a). The pulse-membrane is inserted to the upper part, arranging the scaffold between upper part and pulse membrane (b). The system is locked by the lower part (c) and transferred backwards (d) to the core unit of the bioreactor.

**Figure 4:**
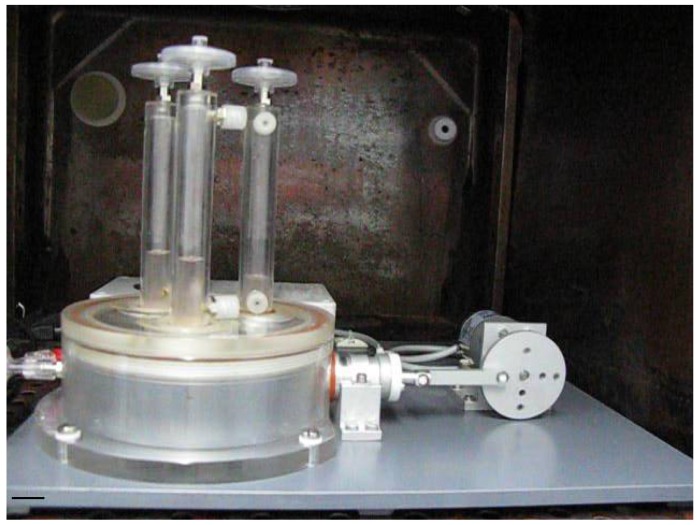
Pulsatile bioreactor operating in a conventional incubator. Comparison of different media, cells and scaffolds are enabled by three media compartments at defined mechanical loadings. Core unit manufacturing from acrylic glass provides optical transparency for macroscopical observation of processes within the unit. Scale bar = 2 cm.

Fixing of seeded scaffolds by clip-systems allows easy assembling, reliable fixing and facilitates sterile operations. Culture medium in the media compartments supplies colonized scaffolds with nutrients. Removal of spent media and filling with fresh media is allowed by luer-lock-connectors at three media compartments. Three media compartments enable the comparison of different media, cells and scaffolds at defined mechanical loadings. The speed controlled gear motor provides frequencies at 1–65 bpm, offering gradually increasing mechanical loadings of the tissue-engineered scaffolds. Manufacturing of the core unit from acrylic glass provides optical transparency for macroscopical observation of processes within the unit. Elements of acrylic glass, stainless steel, Teflon® and silicone are robust and can be gas sterilized. In addition, the bioreactor was designed in a dimension that allows operations in a standard incubator (see Figure 4).

### 3.3. Bioreactor Sterility

Culture medium was checked during the bioreactor run at 24 h and 96 h for signs of contamination by conventional microbiological evaluation methods (streak sample), performed by professionals from the Max-von-Pettenkofer-Institute for Hygiene and Medical Microbiology (University of Munich, Munich, Germany). The results of these examinations showed no microbiological contamination in a period of 96 h, indicating the possibility of dynamic studies under sterile conditions.

### 3.4. Bioreactor Functionality

**Figure 5:**
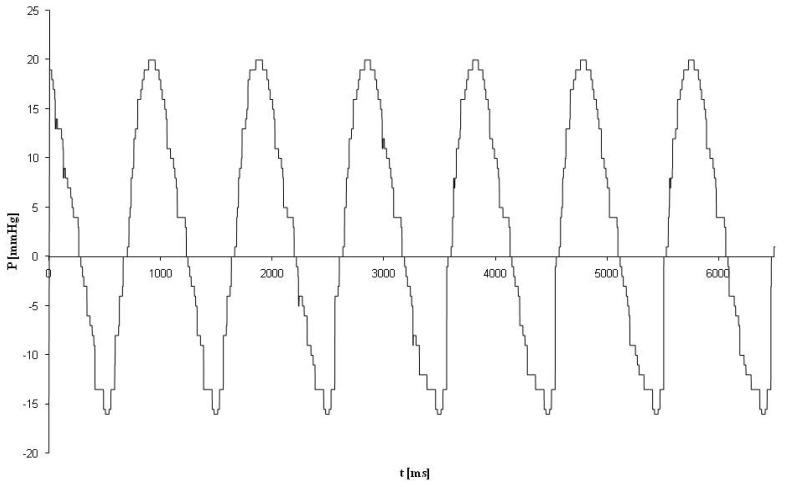
Pressure measurements using a medical pressure sensor in the pressure compartment of the bioreactor. Pressure measurements at 65 bpm showed a uniformly sinusoidal-like pulsation with an overall amplitude of 35 mmHg (positive values represent the applied pressure on seeded scaffolds; negative pressure values represent the relaxation phase).

Bioreactor functionality was macroscopically and sensorically checked. The bioreactor demonstrated a uniformly sinusoidal-like pulsation curve with an overall amplitude of 35 mmHg (positive values represent the applied pressure on seeded scaffolds; negative pressure values represent the relaxation phase) after a frequency of 65 bpm, indicating that the bioreactor performed well (see Figure 5).

### 3.5. Adherence of UCMSC after Mechanical Stress

**Figure 6:**
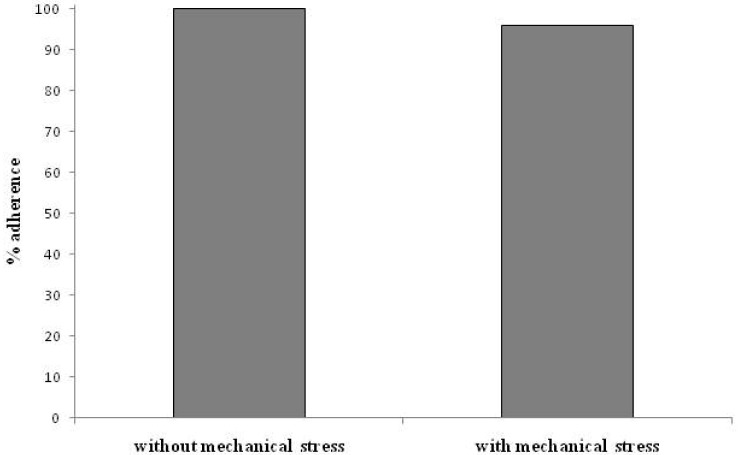
Adherence rate of mesenchymal stem cells derived from umbilical cord tissue (UCMSC) after mechanical stress. The adherence rate of UCMSC after mechanical stress does not differ from the adherence rate of UCMSC without mechanical stress. Results are given as mean values of duplicates.

UCMSC were seeded on titanium-coated CVP to compare the adherence rate without mechanical stress and after mechanical stress for 24 h. The resulting adherence rates are shown in Figure 6. The adherence rate of UCMSC with mechanical stress is similar to the adherence rate of UCMSC without mechanical stress. Lack of mechanical stress can be excluded by pulsating menisci in the media compartments after macroscopic observation.

### 3.6. Morphology of UCMSC after Mechanical Stress

**Figure 7:**
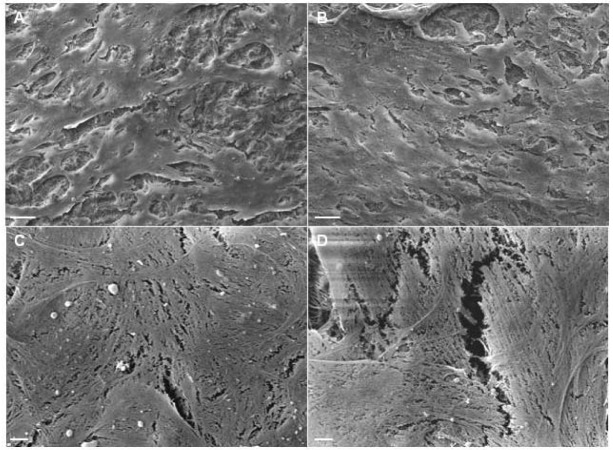
SEM images of UCMSC on titanium coated Cardiovascular Patch (CVP) in the presence or absence of mechanical stress. (**a**) Confluency of UCMSC after mechanical stress is similar to (**b**) confluency of UCMSC without mechanical stress. (**c**) The cell surface of UCMSC after mechanical stress does not differ from (**d**) the surface of UCMSC in the absence of mechanical stress. These are representatives of an experiment in duplicate. Scale bars: A, B = 100 μm (magnification 500×), C, D = 5 μm (magnification 2000×).

The morphology of UCMSC on titanium-coated CVP after mechanical stress for 24 h and in the absence of mechanical stress was analyzed by SEM. As demonstrated in Figure 7, UCMSC confluency after mechanical stress (a) is similar to UCMSC confluency without mechanical stress (b), even at a higher magnification (c) and (d), respectively. The lack of differences in the appearance of mechanically stressed UCMSC and unstressed UCMSC indicates the mechanical integrity of the cellular coating to friction stress. However, the effect of mechanical stress on UCMSC viability, proliferation and differentiation capacity remains to be analyzed in further studies.

In contrast to previously described bioreactors aiming to stimulate contraction of engineered tissue [13,14,15] or a bioreactor for conditioning of cardiovascular patches [16], we focused on the development of a bioreactor to analyze the integrity of cell seeded scaffolds used as a delivery system, to ensure maintenance of stem cells in the infarcted area. Studies of Birla *et al.* using a bioreactor system to deliver stretch forces to bioengineered heart muscle, demonstrate construct stability in response to stretch protocols. However, we solely investigated the effect of friction stress to cellular coatings and are thus, to best of our knowledge, the first to develop a bioreactor for this application.

## 4. Conclusions

We demonstrated the successful development of a novel pulsatile bioreactor for the exposure of cell seeded scaffolds to mechanical stress. Functionality and sterility studies show that the bioreactor performed as expected. Furthermore, preliminary experiments on UCMSC-seeded scaffolds after friction stress reveal the mechanical integrity of the cellular coating. After the *in vitro* evaluation of a stable cellular coating, the next step will be the analysis of functional reconstruction of heart muscle by tissue-engineered constructs in a pre-clinical model.

## References

[1] Morsi Y.S., Yang W.W., Owida A., Wong C.S. (2007). Development of a novel pulsatile bioreactor for tissue culture. J. Artif. Organs.

[2] Wintermantel E., Ha S.W. (2008). Medizintechnik Life Science Engineering.

[3] Barron V., Lyons E., Stenson-Cox C., McHugh P.E., Pandit A. (2003). Bioreactors for cardiovascular cell and tissue growth: A review. Ann. Biomed. Eng..

[4] Shachar M., Cohen S. (2003). Cardiac tissue engineering, *ex-vivo*: Design principles in biomaterials and bioreactors. Heart Fail. Rev..

[5] Bilodeau K., Mantovani D. (2006). Bioreactors for tissue engineering: Focus on mechanical constraints. A comparative review. Tissue Eng..

[6] Chen H.C., Hu Y.C. (2006). Bioreactors for tissue engineering. Biotechnol. Lett..

[7] Goldstein A.S., Christ G. (2009). Functional tissue engineering requires bioreactor strategies. Tissue Eng. Part A.

[8] Dumont K., Yperman J., Verbeken E., Segers P., Meuris B., Vandenberghe S., Flameng W., Verdonck P.R. (2002). Design of a new pulsatile bioreactor for tissue engineered aortic heart valve formation. Artif. Organs.

[9] Engelmayr G.C., Hildebrand D.K., Sutherland F.W., Mayer J.E., Sacks M.S. (2003). A novel bioreactor for the dynamic flexural stimulation of tissue engineered heart valve biomaterials. Biomaterials.

[10] Akins R.E., Boyce R.A., Madonna M.L., Schroedl N.A., Gonda S.R., McLaughlin T.A., Hartzell C.R. (1999). Cardiac organogenesis *in vitro*: Reestablishment of three-dimensional tissue architecture by dissociated neonatal rat ventricular cells. Tissue Eng..

[11] Freed L.E., Vunjak-Novakovic G. (1997). Microgravity tissue engineering. In Vitro Cell Dev. Biol. Anim..

[12] Brown M.A., Iyer R.K., Radisic M. (2008). Pulsatile perfusion bioreactor for cardiac tissue engineering. Biotechnol. Prog..

[13] Akhyari P., Fedak P.W., Weisel R.D., Lee T.Y., Verma S., Mickle D.A., Li R.K. (2002). Mechanical stretch regimen enhances the formation of bioengineered autologous cardiac muscle grafts. Circulation.

[14] Fink C., Ergun S., Kralisch D., Remmers U., Weil J., Eschenhagen T. (2000). Chronic stretch of engineered heart tissue induces hypertrophy and functional improvement. FASEB J..

[15] Zimmermann W.H., Schneiderbanger K., Schubert P., Didie M., Munzel F., Heubach J.F., Kostin S., Neuhuber W.L., Eschenhagen T. (2002). Tissue engineering of a differentiated cardiac muscle construct. Circ. Res..

[16] Sodian R., Lemke T., Loebe M., Hoerstrup S.P., Potapov E.V., Hausmann H., Meyer R., Hetzer R. (2001). New pulsatile bioreactor for fabrication of tissue-engineered patches. J. Biomed. Mater. Res..

[17] Birla R.K., Huang Y.C., Dennis R.G. (2007). Development of a novel bioreactor for the mechanical loading of tissue-engineered heart muscle. Tissue Eng..

[18] Hollweck T., Marschmann M., Hartmann I., Akra B., Meiser B., Reichart B., Eblenkamp M., Wintermantel E., Eissner G. (2010). Comparative analysis of adherence, viability, proliferation and morphology of umbilical cord tissue-derived mesenchymal stem cells seeded on different titanium-coated expanded polytetrafluoroethylene scaffolds. Biomed. Mater..

[19] Seshareddy K., Troyer D., Weiss M.L. (2008). Method to isolate mesenchymal-like cells from Wharton's Jelly of umbilical cord. Methods Cell Biol..

[20] Hartmann I., Hollweck T., Haffner S., Krebs M., Meiser B., Reichart B., Eissner G. (2010). Umbilical cord tissue-derived mesenchymal stem cells grow best under GMP-compliant culture conditions and maintain their phenotypic and functional properties. J. Immunol. Methods.

[21] Campard D., Lysy P.A., Najimi M., Sokal E.M. (2008). Native umbilical cord matrix stem cells express hepatic markers and differentiate into hepatocyte-like cells. Gastroenterology.

[22] Chen M.Y., Lie P.C., Li Z.L., Wei X. (2009). Endothelial differentiation of Wharton's jelly-derived mesenchymal stem cells in comparison with bone marrow-derived mesenchymal stem cells. Exp. Hematol..

